# Relationships between Personal and Collective Place Identity and Well-Being in Mountain Communities

**DOI:** 10.3389/fpsyg.2017.00079

**Published:** 2017-01-31

**Authors:** Igor Knez, Ingegärd Eliasson

**Affiliations:** ^1^Department of Social Work and Psychology, University of GävleGävle, Sweden; ^2^Department of Conservation, University of GothenburgGöteborg, Sweden

**Keywords:** mountain, cultural ecosystem services, place-identity, well-being

## Abstract

The aim was to investigate the relationships between landscape-related personal and collective identity and well-being of residents living in a Swedish mountain county (*N* = 850). It was shown that their most valued mountain activities were viewing and experiencing nature and landscape, outdoor recreation, rest and leisure, and socializing with friends/family. Qualitative analyses showed that the most valued aspects of the sites were landscape and outdoor restoration for personal favorite sites, and tourism and alpine for collective favorite sites. According to quantitative analyses the stronger the attachment/closeness/belonging (emotional component of place identity) residents felt to favorite personal and collective sites the more well-being they perceived when visiting these places. Similarly, the more remembrance, thinking and mental travel (cognitive component of place identity) residents directed to these sites the more well-being they perceived in these places. In both types of sites well-being was more strongly predicted by emotional than cognitive component of place-identity. All this indicates the importance of person-place bonds in beneficial experiences of the outdoors, over and above simply being in outdoor environments.

## Introduction

Identification with landscape as a cultural ecosystem service and its relation to human well-being has been recognized by Millenium Ecosystem Assessment [MA] (2005). Identity, heritage values, spiritual services, esthetic appreciation of natural and cultivated landscapes, recreation, and tourism are the categories of cultural ecosystem services that are provided by landscapes (Millenium Ecosystem Assessment [MA], 2005). The link between identity and well-being has, however, not as yet been fully addressed. Above all, because the concept of ecosystem services is primarily based on natural and economic science paradigms (Daily et al., 2009; Schaich et al., 2010; Tengberg et al., 2012) not including cultural ecosystem services *per se* (Chan et al., 2012; Hernández-Morcillo et al., 2013). Hence, more research is needed especially on the links between biological ecosystem outcomes, cultural landscape issues (Gee and Burkhard, 2010), health and well-being (Sandifer et al., 2015); a concern that is indicated in, for example, the European Landscape Convention [ELC] (2000).

### Landscape-Related Identification

Definitions of landscape include not only objective natural characteristics (Turner, 1989), but also subjective human views, perceptions, identifications and memories (Knez, 2006; Knez and Thorsson, 2008; Lewicka, 2008; Stobbelaar and Pedroli, 2011). We evolve personal and collective ties toward landscapes, meaning that sites encompass not only physical and spatial parameters but also psychological, social, historical, religious, moral, health and cultural connotations (Graumann, 2002; Knez, 2005, 2013, 2016a; Knez et al., 2009; Knez et al., 2013; Lachowycz and Jones, 2013; Gunnarsson et al., 2016; Ode Sang et al., 2016). Culture is to society what memory is to individuals (Triandis, 1994), involving traditions and practices regarding how we perceive and comprehend physical surroundings and ourselves (Canter, 1997; Knez and Thorsson, 2006).

Accordingly, neither the individual nor the collective is placeless (Millenium Ecosystem Assessment [MA], 2005; European Landscape Convention [ELC], 2000). We anchor our existence in physical places, meaning that places serve to “*situate* one’s memorial life” (Casey, 2000 p. 184). In line with this, place-related cognitions have been shown to comprise both personal (Knez, 2006; Taylor, 2010) and collective information (Lewicka, 2008) operating as autobiographical memory aids in self formation (Knez, 2014). This means that natural sites can act as reminders of important experiences and occurrences, by which we uphold and consolidate personal and collective types of identification (Wang, 2008; Wheeler, 2014).

Collective identity is linked to “group membership, group processes and intergroup behavior,” and personal identity is associated with “close personal relationships and idiosyncratic attributes” (Hogg, 2006, p. 463). The type of identity of primary interest here is personal and collective knowledge apportioned across declarative memory as autobiographical memory (Kihlstrom and Klein, 1994); a self-related memory (Conway, 2005) resulting in “feeling that we are re-living our past” (Klein, 2013, p. 3). This type of cognizance is phenomenologically characterized as a life story (Fivush, 2008), involving several context-specific selves (McConnell, 2011; Knez, 2014, 2016b).

One such self is landscape-related identification (Stobbelaar and Pedroli, 2011), a place-related self, containing emotional and cognitive processes accounting for the experience of favorite personal and/or collective places respectively (Knez, 2014). Consistent with, for example, James (1890/1950), Tulving (2002), Conway et al. (2004), Klein et al. (2004), and Knez (2014) suggested a role for the cognitive processes of mental temporality, coherence, correspondence, reflection, and agency, as well as a role for the emotional process of attachment/closeness/belonging (Korpela, 2012) in place-related identification; accounting for the phenomenon of place-related self.

According to Knez (2014) place-related attachment, in agreement with Ainsworth et al. (1978) concept of secure attachment, includes a dimension of closeness/belonging (an emotional component of people-place bonding). Wang (2006) showed that earliest childhood memories were cued by the words mother and surrounding, suggesting an early development of the emotional component of attachment/closeness/belonging to a person and a site. Accordingly, this suggests that our personal and collective favorite places might operate as organizational structures in the autobiographical memory; that is, as chapters in a life story (Thomsen, 2009) clustering our personal and collective memories related to personal and collective favorite places respectively. Furthermore and giving that we remember better events that are emotionally processed than those that are not (Canli et al., 2000), Knez (2014) proposed and showed that a favorite place might be easier recalled due to its emotional information.

The model of a place-related self is conceptualized and operationalized in line with the view that: (1) “we are what we remember,” and (2) a stable and healthy self encompasses processes of mental temporality, coherence, correspondence, reflection, agency, and attachment/closeness/belonging (Klein et al., 2004; Wang, 2006). This suggests that a place-related self is a higher-order construct (Stajkovic, 2006) capturing basic psychological processes grounding the relationship between a physical place and the self. Thus, the place-related self is conceptualized as a knowledge structure (Kihlstrom and Klein, 1994) resulting in a *personal* autobiographical experience of “*my* place” (Knez, 2014) as opposed to, for example, the construct of ecological self, accounting for the link between an environmentally responsible behavior and a world view (Bragg, 1996). (See also, for example, Neisser (1988) and Leary and Tangney (2003) for a discussion about different types of self and identity constructions in psychology.)

In the words of Knez (2014, p. 186): “…physical places and time *position* -anchor- one’s reminiscence by forming psychological person-place ties, emotional and cognitive bonds that conduct the psychological agent toward physical places and time as *the* organizing formats for its personal memory… A place-related self is, thus, assumed to be a substructure of the self, emerging when we cogitate about our lives, when our self-representations are online, triggering streams of *noeses*-ways of knowing about ourselves.” In consequence, this brings an additional theoretical position regarding the phenomenon of person-place ties than the more traditional ones of sense of place (Jorgensen and Stedman, 2001), place attachment (Scannell and Gifford, 2010), and place identity (Droseltis and Vignoles, 2010) considering, for example, person-place-bonding-dimensions of identity and dependence to be integral parts of attachment (Brown and Raymond, 2007) or as separate dimensions (Jorgensen and Stedman, 2006).

In view of that, and as an example, by revealing a piece of my personal story, I have across *time* evolved an *attachment/closeness/belonging* toward a small place (Axmar) on the Swedish east coast (*a place of mine*) where I have a cottage. This place and its surrounding sea landscapes have grounded my *landscape-related identification* (conceptual and personal knowledge of me as cottage owner and user of its surroundings, including different types of emotional, cognitive and behavioral experiences). *Reflecting* upon these involvements of mine (*agency*) I remember the date and the time of day (inner *temporality*) when I bought this cottage (*coherence* in my landscape identification). As a consequence of all this and at this precise moment, I am thinking of the coming weekend when I will visit this site for some rest, leisure and outdoor recreation (an accurate *correspondence* with my ongoing landscape identification).

This proposes that we do not only cognize (processes of coherence, correspondence, reflection, inner temporality, and agency) about places in our life but we also emotionally invest (process of attachment/closeness/belonging) in these places (Marris, 1982). In other words: “Natural or semi-natural features of the environment are often associated with the identity of an individual, a community, or a society. They provide experiences shared across generations, as well as settings for communal interactions important to cultural ties” (Daniel et al., 2012 p. 8814). This suggests that both natural and cultural values are important for our personal and collective memory and heritage (Lowenthal, 2005; Aplin, 2007). As pointed out by Erll (2011, p. 13): “Acts of cultural remembering seem to be an element of human’s fundamental anthropological make-up, and the history of creating shared heritage and thinking about memory can be traced to antiquity, for example to Homer, Plato, and Aristotle.”

### Landscape-Related Well-Being

On his tour of Lapland (northern part of Sweden) in 1732, Carl von Linné suggested a link between the natural scenery and well-being. In ancient Greece and Rome, as well as in many other cultures and religions, an archetypical landscape (such as the Biblical Garden of Eden) was/is associated with a supreme type of life (Thompson, 2011). Transcendent experiences (positive affect associated with a sense of timelessness) have also been shown to associate with natural environments (Laski, 1961; Williams and Harvey, 2001; Park et al., 2011). This therapeutic use of nature, therapeutic associations between nature and well-being including feelings of solitude and aesthetical values (Russell, 2012) are of course also portrayed in works of fiction, such as in Thomas Mann’s *The Magic Mountain* containing impressions and images of “pure Alpine air and magnificent mountain landscapes” (Gesler, 2000, p. 126), and in self-biographical reflections of for example “Throughout my childhood, I always felt drawn to a mountain in the heart of Alaska which the Athabaskan Indians call Denali, The Great One … Now, years later, I still recall the many transcendent experiences during my ascent of this tallest mountain in North America.” (Hébert, 2014, p. 27), and “When I am lonely the mountains call me” (Griffin-Pierce, 1997 p. 1).

In line with this, many studies have indicated health and well-being benefits of the natural environment for humans (Lachowycz and Jones, 2013; Bratman et al., 2015), such as positive effects of nature on physiological, psychological, social and cultural variables (Abraham et al., 2010; Bowler et al., 2010; Hartig et al., 2011; Carrus et al., 2015; Sandifer et al., 2015). Theoretical models have in particular drown on emotional and aesthetical (Ulrich, 1983), and cognitive explanations for nature-related restoration (Kaplan, 1995). Some have also indicated geographical inequalities in health (e.g., Bolam et al., 2006). In parallel with Millenium Ecosystem Assessment [MA]’s 2005 definition of cultural ecosystem services and their relation to human well-being several findings have shown that different levels of nature engagement (viewing nature, presence of nature, participation and involvement with nature) provide health benefits to humans (Pretty, 2004; Pretty et al., 2005). However, previous research (e.g., Hartig et al., 2011; Clayton, 2012) does not tell us much about the link between identity and memory we address to personal and collective favorite places and the well-being we experience when visiting these sites (e.g., Tuan, 1977; Gallagher, 1994; Knez, 2014).

In addition, the relation between the self/identity and the nature has been previously operationalized into several types of scales measuring phenomena of, for example, inclusion of nature in the self (Schultz, 2002), connectedness to nature (Mayer and Frantz, 2004), and connectivity to nature (Dutcher et al., 2007). Some previous research has moreover indicated relationships between restorative sites and person-place bonding (Korpela, 1989; Scannell and Gifford, 2010; Pretty et al., 2015), but no studies, as far we know, have investigated the links between landscape-related personal and collective identity (cultural ecosystem services as defined by Millenium Ecosystem Assessment [MA], 2005) and well-being in residents living in a mountain county; as provocatively stated by Grêt-Regamey et al. (2012) in their review of 155 studies on mountain ecosystem services: “Mountain Ecosystem Services: Who Cares?”

### Aims and Hypotheses

The aim of this study was, thus, to investigate residents’ mountain landscape-related personal and collective identity (involving emotional and cognitive processes) and well-being. In line with previous research on the positive relations between nature and health and well-being (Abraham et al., 2010; Bowler et al., 2010; Hartig et al., 2011; Sandifer et al., 2015) and the Millenium Ecosystem Assessment [MA] (2005) prediction of a positive link between cultural ecosystem services of identity and well-being, our general prediction was that place-related identifications with mountains will positively predict well-being associated with visiting these sites.

This suggests that mountain experiences might be viewed as elements of a person’s identity (Borrie and Birzell, 2001; Clayton, 2003), meaning that favorite places might become intertwined with the self (Tuan, 1977; Knez, 2014). Accordingly, the self might practically recommend itself to visit favorite places to increase well-being in a self-reflective and self-regulating way (Korpela, 1989, 1992; Korpela and Hartig, 1996; Korpela et al., 2001; Knez, 2006), proposing that revisiting a favorite place might, in consequence, be considered as an affect-regulation strategy. Parkinson and Totterdell (1999) have indeed shown that visiting-a- favorite-place is classified as an affect-regulation strategy. All this is in line with the theory of self-regulation, suggesting that we, by forethought (Bandura, 1991), monitor, regulate and advocate our behaviors proactively such as to minimize negative affect and to increase positive affect and health behavior (Carver and Scheier, 1990; Heatherton, 2011; Mann et al., 2013).

In addition, residents’ reports of mountain-related activities were also collected, as well as perceptual and aesthetical values, cultural and historical identifiers associated with their personal and collective favorite sites. This was done because nature-related bonding involves multiple ways of, and activities connected with, appreciating nature (Hammitt et al., 2006; Brown and Raymond, 2007; Brügger et al., 2011; Hayden et al., 2015) providing health benefits (Pretty, 2004; Pretty et al., 2005). In addition, activities such as esthetic appreciation of natural landscapes, and recreation are categories of cultural ecosystem services that are provided by landscapes (Millenium Ecosystem Assessment [MA], 2005).

Finally and line with (1) Canli et al. (2000) and Phelps (2006) indicating that emotion may enhance and modulate better retention in autobiographical memory, (2) that emotion may regulate intrinsic psychological processes (Gross, 2010), and (3) that the emotional component may precede the cognitive one in place-identification because a favorite place might be easier recalled due to its emotional information (Knez, 2014), we predicted that emotional bonds would be stronger predictors of well-being than cognitive bonds in relation to both personal and collective favorite places.

## Materials and Methods

### Study Area

**FIGURE 1:**
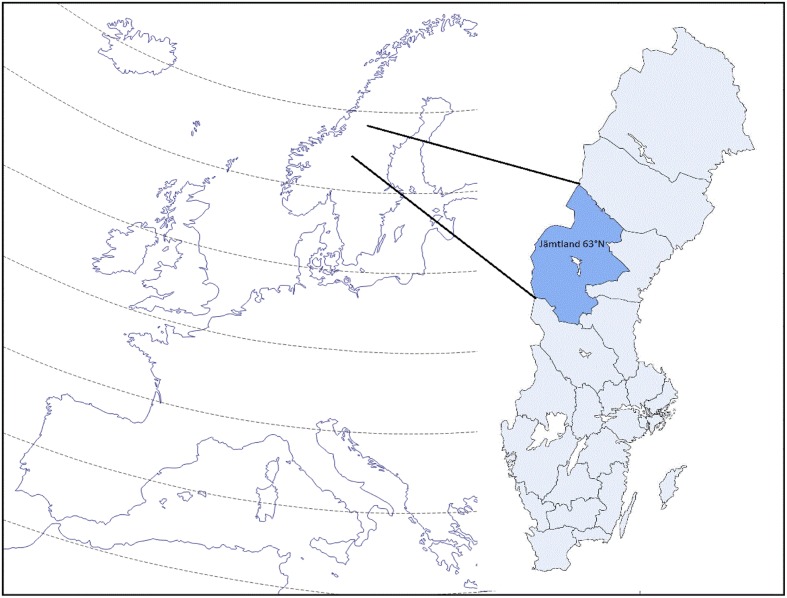
**The map of Europe and Sweden, with Jämtland county**.

The county of Jämtland is located at a latitude of 63°N in the northwest part of Sweden with the Scandinavian Mountains bordering Norway (see **Figure 1**). With its area of 49,443 km^2^, it is the third largest county in Sweden and comparable to the size of the Netherlands.

Jämtland has a diverse geography with old forests to the east, a rolling landscape in the central areas, and alpine massifs up to 1,797 meters above sea level (MASL) to the west. The climate is harsh with predominantly west winds and monthly mean temperatures of -8°C in January and 13.5°C in July for the city of Östersund. The county has been an important tourism destination since the opening of the railway to Norway in the late 19th century. It also includes 12 Sami villages with the right to carry on reindeer husbandry.

### Sample

A total of 2,700 households, proportionally and randomly distributed across eight municipalities, identified from a population register, were sent a “mountain survey.” Participants were not offered any incentive for taking part in the survey, which involved 12 sections including both quantitative and qualitative questions. Data on mountain-related activities, values, cultural and historical identifiers, personal and collective place-identity and well-being will be reported in this study. Finally, the survey was conducted in accordance with the ethical guidelines of the University of Gothenburg Sweden, in charge of the project; therefore, not reviewed and approved by any special ethic committee.

### Procedures, Response Rate and Demographic Statistics

Surveys were distributed and returned by mail. After two contacts (a reminder was sent a week later) 850 responses (32%) were achieved proportionally distributed across the eight municipalities, involving 51.7% women and 48.3% men. 44.9% of the participants were 18–55 years old and 50.1% 56–80 years old. The participants’ mean residence time, living in Jämtland County, was 40.6 years (ranging from one to 79 years). Their educational background was distributed across three types of education: elementary education (19.4%), upper secondary education (42.1%) and university education (38.4%). Most of participants were employed (58.5%) or retirees (30.1%). The questionnaire was fully anonymous.

### Measures

*What do you do when you are in the Jämtland County mountains?* Participants were asked to estimate the *frequency* (how many times per year) of performing each one of the eighteen activities: Spirituality; Work; Berry and mushroom picking; Festival, exhibitions, markets; Outdoor recreation; Bird and wildlife watching; Health experiences; Hunting, fishing; Consumption, trade; Museums, historical sites; Nature and landscape; Fun and nightlife; Snowmobiling; Sports, sports activities; Socializing with friends/family; Tradition (family, culture); Education; Rest, leisure; Adventure. They were also asked to estimate the *importance* of each type of activity on a 7-point scale, ranging from 1 (little/not at all important) to 7 (very important). This measure was included because nature-related bonding involves multiple ways of appreciating nature (Hammitt et al., 2006; Brown and Raymond, 2007; Brügger et al., 2011). Additionally, we asked participants to estimate *during which time of the year and how often they engaged in these activities*, on a scale ranging from 1 to 5: daily (1); one to several times a week (2), one to several times a month (3), one to several times per season (4); more rarely/never (5).

*Which five Jämtland County mountain sites are most important for you personally? By this we mean places that are your favorites; places which increase the understanding of who you are?* This was a qualitative data question; participants were asked to write down the names of these places (or indicate on a map that was included in the survey). They were further asked to *select one of the places which is most important for you personally (your favorite site) and answer the following questions about it: What do you value most about this place? What are the site’s cultural and historical identifiers?* Participants were asked to write down their responses. All this was repeated for: *Which five Jämtland County mountain sites are important for you living in Jämtland; that is, places that enhance the understanding of the County’s identity?* (Note: plural form of “you” in Swedish is “er” and singular form is “dig”; hence, different words that connote directly to collective vs. personal dimensions of “you.”)

This measure was included because collective memory is established both as internal (memory of the individual) and external (culturally and historically shared memory of the collective) reminiscence (Halbwachs, 1941/1992), revealed through individual autobiographies (Rigney, 2005; Assmann, 2008; Manier and Hirst, 2008). Previous research has also shown that places’ cultural and historical dimensions might create a sense of continuity (Devine-Wright and Lyons, 1997; Rishbeth and Powell, 2013), as well as be related to place ties (Low, 1992; Lewicka, 2005).

#### Place-Identity

This measure involves an autobiographical emotional and a cognitive component comprising 10 statements (Knez, 2014). *Emotional component* (processes of attachment/closeness/belonging; in the present study, with a Cronbach alpha of 0.91): “I know the place very well.”; “I miss it when I’m not there.”; “I have strong ties to the place.”; “I am proud of the place.”; “The place is a part of me.” *Cognitive component* (processes of coherence, correspondence, mental temporality, reflection and agency; in the present study, with a Cronbach alpha of 0.92): “I have had a personal contact with this place over a long period.”; “There is a link between the place and my current life.”; “I can travel back and forth in time mentally to this place when I think about it.”; “I can reflect on the memories attached to this place.”; “These thoughts about the place are part of me.” Participants were asked to respond to these statements on a 7-point scale ranging from 1 (completely disagree) to 7 (completely agree). This was the personal place identity measure. For the collective measure we changed the pronoun “I” to “we (living in the county of Jämtland).”

#### Well-Being

Participants were asked to respond to ten statements from “The WHO (10) well-being index” (Bech et al., 1996), measuring their place-related well-being. They responded to the question of *when I’m on the site, I feel*: “Sad and down” (R); “Calm and relaxed”; “Energetic, active and enterprising”; “Relaxed and refreshed”; “Happy and pleased with my personal life”; “Satisfied with my living situation”; “I live the life I want to live”; “Inspired to deal with today’s work”; “I can cope with serious problems or changes in my life”; “That life is full of interesting things.” Furthermore, the 4-point scale from the original measure was rearranged yielding a 7-point scale, ranging from 1 (completely disagree) to 7 (completely agree), with a Cronbach alpha of 0.91.

### Design and Analyses

Both qualitative (inductive thematic organization of the open question answers) and quantitative data (ANOVA and regression) analyses were performed, see Section “Results.” The qualitative, inductive analysis “begins with specific observations and builds toward general patterns. Categories or dimensions of analysis emerge from open-ended observations as the inquirer comes to understand patterns that exist in the phenomenon being investigated” (Patton, 2002, pp. 55–56).

## Results

The qualitative and quantitative data analyses are reported below in three sections: “mountain activities and season,” “personal sites in mountains and well-being,” and “collective sites in mountains and well-being.”

### Mountain Activities and Season

**FIGURE 2:**
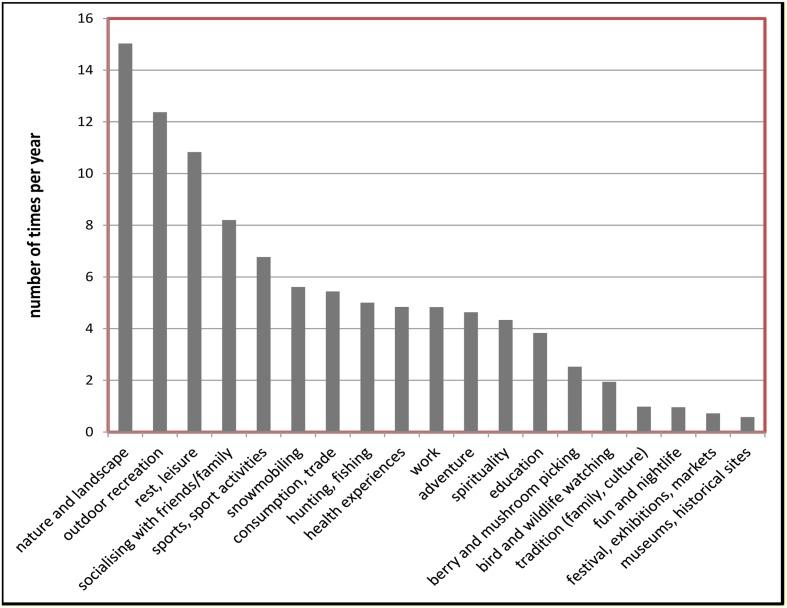
**Frequency (number of times per year) of nineteen types of mountain activities**.

**FIGURE 3:**
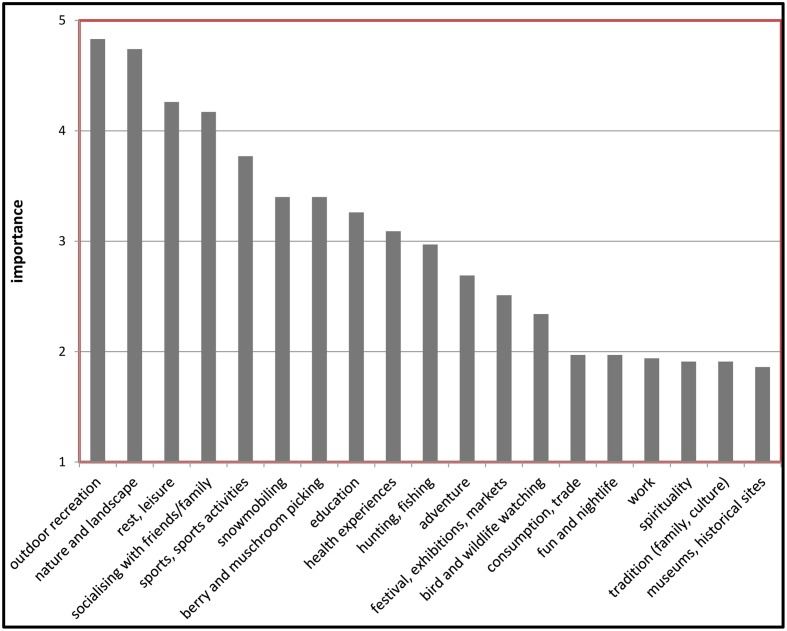
**Mean importance of nineteen types of mountain activities**.

As can be seen in **Figure 2**, descriptive statistics showed that the four most frequent (number of times per year) mountain activities performed by the residents were “outdoor recreation” (*M* = 15.3, *SD* = 55.15), “nature and landscape” (*M* = 12.37, *SD* = 40.03), “rest and leisure” (*M* = 10.83, *SD* = 40.79), and “socializing with friends/family” (*M* = 8.2, *SD* = 30.86). These activities were also valued to be the four most important ones (see **Figure 3**); “outdoor recreation” (*M* = 4.83, *SD* = 2.27), “nature and landscape” (*M* = 4.74, *SD* = 2.34), “rest and leisure” (*M* = 4.26, *SD* = 2.54), and “socializing with friends/family” (*M* = 4.17, *SD* = 2.58). Thus, participants were shown to be consistent in judging frequency of their activities with their estimations of the importance of these activities.

Mountain activities were furthermore shown to be performed mostly during winter (*M* = 3.2, *SD* = 1.33) and least during summer (*M* = 3.46, *SD* = 1.38). In other words, residents were almost “one to several times a month” (3 on a 5-point scale) in the mountains and during summer they were there in between “one to several times a month” and “one to several times during the season” (4 on a 5-point scale) in the mountains.

### Personal Sites in Mountains and Well-Being

The qualitative data analysis (participants’ own words in “…”) showed that 86% of residents pointed out 176 favorite sites in the mountains as the personally most important ones; 50% of these were mentioned only once. This indicates that half of the residents had *settled an own* favorite spot in the mountains. Most of these sites were in Åre (27%), Ovik (18%), West Jämtland (17%), West Härjedalen (13%), and Vemdalen/Lofsdalen (12%) mountain areas. Based on three criteria (geology, communication and cultural heritage) these areas were characterized as follows:

(1) *Åre* mountains have been of great geological importance for the understanding of the plate tectonic theory. This is because shale layers at Mount Åreskutan (1420 MASL) are reversed which means that older layers are above younger ones. Åre has nearly 800,000 guest nights a year, mainly during the winter season December to April.
(2) Mountains in the *Ovik area* consist of quartzite and reach up to 1,400 MASL. The area is located close to the city of Östersund and its airport. The area is popular for skiing, hiking, fishing and hunting and includes two Natura 2000, EU nature protection areas.
(3) *West Jämtland* has a plateau character due to the huge overshoot of resistant bedrock consisting of shales rich in quartz, gneiss and amphibolies. Most of the mountains, however, reach up to 1,500 MASL. The area is appreciated for being a wilderness and inaccessible although it hosts Sami reindeer husbandry.
(4) Mountains in West Härjedalen reach up to 1,300 m MASL and the bedrock consists of resistant shales rich in quartz, gneiss and amphibolites. The tourist destination markets itself as Scandinavia’s largest ski area.
(5) Mountains in *Vemdalen/Lofsdalen* reach up to 1,200 MASL and consist of a quartzite bedrock that was formed during the Cambrian and Silurian, 542-416 million years ago. The area hosts the fourth largest winter sports destination in Sweden as, well as Sami villages and several mountain pastures of which some are farmed and some are classified as Natura 2000 EU nature protection areas.

*What do you value most in this place?* The five most valued attributes were: *Landscape* 19.5% (“beautiful, wide vistas, views, terrain, towering mountain”); *Outdoor restoration* 13.5% (“relaxation, recreation, spirituality, freedom, silence, peace, and quiet”); *Ease of use* 13.5% (“near where I live, day trips are possible, ski-in-ski-out”); *Alpine* 10% (“skiing and slopes/ski resorts”); *Undeveloped* 7% (“untouched, genuine mountain environment, wilderness, solitude, few people”).

*What are the site’s cultural identifiers?* The five most frequent categories were: *Historic buildings* 14.5% (“settlements, settlements and churches”); *Sami culture* 13% (“nest sites, chapels, reindeer husbandry”); *Stories/local history* 10% (“about villages, towns, accommodation”); *Natural sites* 9% (“waterfalls, rivers, lakes, rivers, mountains, caves”); *Industrial history* 8% (“mines, mills, sawmills”).

*What are the place’s historical identifiers?* The five most frequent categories were: the *Carolean March* (a historical war-related march across the Jämtland mountains in1718) 19.5%; *Stories/local history* 13%; *Industrial history* 8.5%; *History of tourism* 6.5% (“visitors arriving by plane, spa resort, hotels and exploitation”); *Infrastructure* 6.5% (“rail, roads, bridges, aerial tramway”).

#### Place-Related Identity and Well-Being

The quantitative data (regression) analysis (including emotion and cognitive components as predictors, and well-being as criterion variable) showed that both components (emotion + cognition) of personal place-identity were positively associated with well-being (see **Table 1**). This means that the stronger the attachment/belonging/closeness (emotional component) residents felt to a favorite *personal* site in mountains the more well-being they perceived in that place. Similarly, the more remembrance, thinking and mental travel (cognitive component) residents directed to this site the more well-being they perceived in that place. However, and as predicted the emotion-wellbeing link compared to the cognition-wellbeing link was stronger (see Beta statistics in **Table 1**).

**Table 1:** Regression statistics for the relation between *personal* place-identity (predictors: components of emotion and cognition) and well-being (criterion).

*R*	Beta	*SE*	df	MS	*F*	Sig.	*t*	Sig.
0.39	0.45 (emotion)	0.04	2,756	179.3	244.4	0.00	8.19	0.00
	0.20 (cognition)	0.03					3.71	0.00


### Collective Sites in Mountains and Well-Being

The qualitative data analysis (participants own words in “…”) showed that 78% of respondents pointed out 91 collectively important sites in Jämtland; 70% of these were mentioned more than once. Similarly, as above for personal sites, the majority of sites were in the Åre (29%), Ovik (16%), Vemdalen/Lofsdalen (15%), West Härjedalen (13%), and West Jämtland (12%) mountains.

*What do you value most in this place?* The five most valued attributes were: *Tourism*, 21% (“tourism, employments, branding of the county”); *Alpine* 16%; *Ease of use* access 10.5%; *Landscape* 10.5%; Outdoor restoration 5.5%.

*What are the site’s cultural identifiers?* The five most frequent categories were: *Tourism* 13%; *Natural sites* 11.5%; *Historic buildings* 11.5%; *Stories/local history* 9%; *Outdoor life* 7.5% (“hiking, skiing, mountain stations, mushroom-picking, berries, hunting and fishing”).

*What are the place’s historical identifiers?* The five most frequent categories were: the *Carolean March* 30%; *History of tourism* 10%; *Stories/local history* 9%; *Sports* 7.5% (“World Cup, sports contests, sports events/stories); *Infrastructure* 6.5%.

#### Place-Related Identity and Well-Being

The quantitative data (regression) analyses (including emotion and cognitive components as predictors, and well-being as criterion variable) showed similar results as above; namely, that both components (emotion + cognition) of collective place-identity were positively related to well-being (see **Table 1**). Thus, the stronger the attachment/belonging/closeness (emotional component) residents felt to a favorite *collective* site in mountains the more well-being they perceived in that place. Similarly, the more remembrance, thinking and mental travel (cognitive component) residents directed to this site the more well-being they perceived in that place. However, and as predicted emotion component of place identity was a stronger predictor of well-being than cognition component of place-identity (see Beta statistics in **Table 2**).

**Table 2:** Regression statistics for the relation between *collective* place-identity (predictors: components of emotion and cognition) and well-being (criterion).

*R*	Beta	*SE*	df	MS	*F*	Sig.	*t*	Sig.
0.34	0.45 (emotion)	0.04	2,668	183.5	170.3	0.00	9.42	0.00
	0.17 (cognition)	0.04					3.49	0.00


## Discussion

The aim was to investigate the relationships between landscape-related personal and collective identity and well-being of residents living in a mountain county. Residents’ reports of mountain-related activities were also collected, as well as perceptual and aesthetical values, cultural and historical identifiers associated with their personal and collective favorite sites in mountains.

It was shown that residents’ most frequent and most important activities, when visiting the mountains, were related to “outdoor recreation,” “nature and landscape,” “rest and leisure,” and “socializing with friends/family.” This is in line with previous research on self-regulation showing that people are active in using nature-related sites for emotional release and relaxation (Korpela, 1989, 1992; Korpela and Hartig, 1996; Knez, 2006), as well as for aesthetical (Brown and Raymond, 2007) and social (Hammitt et al., 2006) values; suggesting that nature engagement generate health benefits (Pretty, 2004; Pretty et al., 2005). Additionally, these behaviors were mostly performed during the winter/spring and least during the summer, due to the deep-rooted winter-outdoor culture in northern parts of Sweden.

Concerning *personal* favorite sites, 50% of residents were shown to settle on an *own* favorite site. In agreement with the above results and reasoning (Korpela, 1989, 1992; Korpela and Hartig, 1996; Hammitt et al., 2006; Knez, 2006; Brown and Raymond, 2007), the most valued attributes related to these favorite sites were aesthetical values of landscape and outdoor restoration experiences. The cultural and historical identifiers involved both built (settlements, nest sites, mills, bridges and roads) and natural dimensions (waterfalls, rivers, lakes, caves) as well as reindeer husbandry and local stories/history associated with these cultural and historical themes. This is in accord with previous research showing that places’ cultural and historical dimensions may create a sense of continuity (Devine-Wright and Lyons, 1997; Rishbeth and Powell, 2013; Wheeler, 2014) and be linked to people-place ties (Low, 1992; Lewicka, 2005), suggesting that “legacies we inherit stem both from nature and from culture.” (Lowenthal, 2005, p. 81).

It was also shown that the stronger the attachment/closeness/belonging residents felt to a favorite *personal* site in mountains the more well-being they perceived in that place. Similarly, the more remembrance, thinking and mental travel residents directed to this site the more well-being they perceived in that place. Thus both emotional and cognitive components of place-identification (Knez, 2014) were positively related to well-being. However, the emotion-wellbeing compared to cognition-wellbeing link was stronger. This is in line with our prediction that a favorite place might be easier cognitively operated due to its emotional content (Canli et al., 2000; Phelps, 2006; Gross, 2010; Knez, 2014). It is also, in general terms, in accordance with previous research suggesting positive relations between nature and health and well-being and between cultural ecosystem services of identity and well-being (Millenium Ecosystem Assessment [MA], 2005; Bowler et al., 2010; Abraham et al., 2010; Hartig et al., 2011; Lachowycz and Jones, 2013; Carrus et al., 2015; Sandifer et al., 2015).

This indicates that mountain experiences might be a part of respondents’ place-related self (Knez, 2014). The fact that respondents revisit these most important favorite places in mountains suggests that these sites are in a sense recommended by the self in an affect- (Parkinson and Totterdell, 1999) and self-regulating way (Korpela, 1989, 1992; Korpela and Hartig, 1996; Knez, 2006). This is done in order to increase positive affect and health behaviors (Carver and Scheier, 1990; Mann et al., 2013); especially, because the emotional component of the place-related self was shown to link strongly to well-being (Gross, 2010). According to Parkinson and Totterdell (1999) a favorite place operates as an affect regulation strategy, suggesting that we advise ourselves proactively (Bandura, 1991) to increase positive affect and health behavior (Carver and Scheier, 1990; Heatherton, 2011; Mann et al., 2013) by going to a favorite place.

Concerning *collective* favorite places, the most valued attributes related to these sites were related to tourism and alpine activities; which is in accordance with the county’s branding. The cultural and historical identifiers involved mostly tourism and outdoor life related dimensions as well as sport events. This suggests an episodic and semantic collective reminiscence, including a combination of shared collective experience (narrative) with “lived” (formed between generations) and “distant” (communicated by institutions) semantic memory (Manier and Hirst, 2008). Furthermore, and with regard to personal favorite sites, the stronger the attachment/closeness/belonging residents felt to a favorite *collective* site the more well-being they perceived in that place. Similarly, the more remembrance, thinking and mental travel residents directed to this site the more well-being they perceived in that place. Thus, collective favorite places were also advised, forethought (Bandura, 1991), by the self to revisit, to increase well-being in a self-regulating way (Parkinson and Totterdell, 1999); but as for personal sites the emotion-wellbeing compared to cognition-wellbeing link was stronger.

## Conclusion

Our results have shown that mountain county residents have individually and collectively pinpointed their favorite sites in mountains to which they have evolved emotional and cognitive bonds, meaning that these locations are part of their personal and collective memory and their life-story (Knez, 2014). When visiting these places they enjoy outdoor recreation/restoration, viewing and experiencing nature and landscape, rest and leisure, and perceive higher levels of well-being; thus, implying a “healthy nature healthy people” relation (Maller et al., 2005). A stronger relationship between the emotional than the cognitive bond of place-identity with well-being was, however, reported, indicating that emotion may enhance, modulate and regulate better these intrinsic psychological processes (Canli et al., 2000; Phelps, 2006; Gross, 2010). All this is in line with previous research indicating (1) positive associations between nature and human health and well-being (Pretty, 2004; Hartig et al., 2011), (2) transcendent experience in nature involving positive affect (Williams and Harvey, 2001), and (3) that cultural ecosystem services of landscape-identifications may enhance well-being (Millenium Ecosystem Assessment [MA], 2005). Finally, what are the practical implications of the results obtained? Our results highlight the importance of person-place bonds not only to urban green spaces (e.g., Gunnarsson et al., 2016; Ode Sang et al., 2016), but also to rural sites and their inhabitants. Given that going to a favorite place is a type of affect-regulation strategy that we use in order to monitor and improve our feelings (Parkinson and Totterdell, 1999), we conclude with Carl von Linné’s self-biographical reflections from the year of 1732 (Linné, 1811, pp. 109–110): “As soon as I reached the mountains I felt reborn and that a heavy burden had been lifted from me.”

## Author Contributions

All authors listed, have made substantial, direct and intellectual contribution to the work, and approved it for publication.

## Conflict of Interest Statement

The authors declare that the research was conducted in the absence of any commercial or financial relationships that could be construed as a potential conflict of interest.

The reviewer ER and handling Editor declared their shared affiliation, and the handling Editor states that the process nevertheless met the standards of a fair and objective review.
